# Target-Guided Isolation of Progenitors of 1,1,6-Trimethyl-1,2-dihydronaphthalene (TDN) from Riesling Wine by High-Performance Countercurrent Chromatography †

**DOI:** 10.3390/molecules27175378

**Published:** 2022-08-23

**Authors:** Recep Gök, Pia Selhorst, Mats Kiene, Theresa Noske, Michael Ziegler, Ulrich Fischer, Peter Winterhalter

**Affiliations:** 1 Institute of Food Chemistry, Technische Universität Braunschweig, Schleinitzstrasse 20, 38106 Braunschweig, Germany; 2 Institute for Viticulture and Oenology, Dienstleistungszentrum Ländlicher Raum (DLR) Rheinpfalz, 67435 Neustadt an der Weinstraße, Germany

**Keywords:** 1,1,6-trimethyl-1,2-dihydronaphthalene (TDN), glycosides, aroma precursors, isolation, petrol note, kerosene off-flavor, carotenoids, Riesling, sunlight exposure, countercurrent chromatography

## Abstract

High-performance countercurrent chromatography (HPCCC) was used for the target-guided isolation of precursors of 1,1,6-trimethyl-1,2-dihydronaphthalene (TDN) from Riesling wine. In separated HPCCC fractions of an Amberlite^®^ XAD^®^-2 extract obtained from a German Riesling, TDN-generating fractions were identified by the acid-catalyzed hydrolysis of the progenitors at pH 3.0 and subsequent HS-GC-MS/MS analysis. The presence of multiple TDN-generating precursors in Riesling wine could be confirmed. From polar HPCCC fractions (11–13 and 14–16), 3,4-dihydroxy-7,8-dihydro-β-ionone 3-*O*-rutinoside and 3,4-dihydroxy-7,8-dihydro-β-ionone 3-*O*-β-d-glucopyranoside were isolated as major TDN-precursors at a sufficient amount for structure elucidation by NMR spectroscopic studies. In the medium polar HPCCC factions (27–35), enzymatic hydrolysis liberated the aglycones 3-hydroxy-β-ionone and 3-hydroxy-TDN in minor amounts. In further less polar TDN-generation fractions (36–44 and 45–50), glycosidic progenitors were absent; instead, a minor TDN formation most likely from non-conjugated constituents was observed.

## 1. Introduction

Riesling is a prominent white wine variety, especially in Germany. Excessive sunlight exposure and high temperatures during the cultivation of Riesling grapes, as well as the prolonged bottle storage of Riesling wines, can lead to a formation of the C_13_-norisoprenoid hydrocarbon 1,1,6-trimethyl-1,2-dihydronaphthalene (TDN, **4**). At low concentrations, TDN may be considered as a typical contributor to the “bottle aged bouquet” of Riesling wine. In higher concentrations, however, it causes an undesirable “petrol or kerosene” off-flavor note [1]. In particular, Riesling wines grown under warmer climatic conditions with high sunlight exposure are affected, and TDN levels up to 255 µg/L have been reported, e.g., for Australian Rieslings [2]. Regarding the flavor threshold of TDN, the originally reported threshold of 20 µg/L [1] was recently adjusted by Sacks et al. [3] and Ziegler et al. [4]. The newly determined detection threshold was found to be 10 times lower, i.e., in the range of 2–2.3 µg/L in the wine medium. Due to global warming, TDN levels in German Rieslings are also increasing [5], and measures to reduce the risk of an off-flavor development are becoming an important issue in current oenological and viticultural research in Germany. Traditionally, Germany is the largest Riesling producer in the world and cultivates more than a third (24,000 ha) of the entire global vineyard area (64,000 ha) planted with Riesling [6].

Genuine progenitors of TDN are non-volatile glycoconjugates consisting of carotenoid metabolites with thirteen carbon atoms (C_13_-norisoprenoids) conjugated to mono- or disaccharides that accumulate during grape development after veraison until maturity. The grape glycosides are transferred into must and, during wine maturation and storage, a slow hydrolytic release of TDN under the acidic pH conditions in wine takes place. For this reason, TDN is only detectable in traces in grapes, grape must and young wines, but slowly increases during the first years of storage [1,7,8]. The occurrence of TDN and its precursors is not limited to grapes. TDN precursors have already been detected in various natural sources, such as quince fruit (*Cydonia oblonga* Mill.) [9], passion fruit (*Passiflora edulis* Sims) [10] and red currant leaves (*Ribes rubrum* L.) [11], as well as apple (*Malus domestica*) [12] and Riesling leaves (Vitis vinifera cv. Riesling), respectively [13].

**Figure 1:**
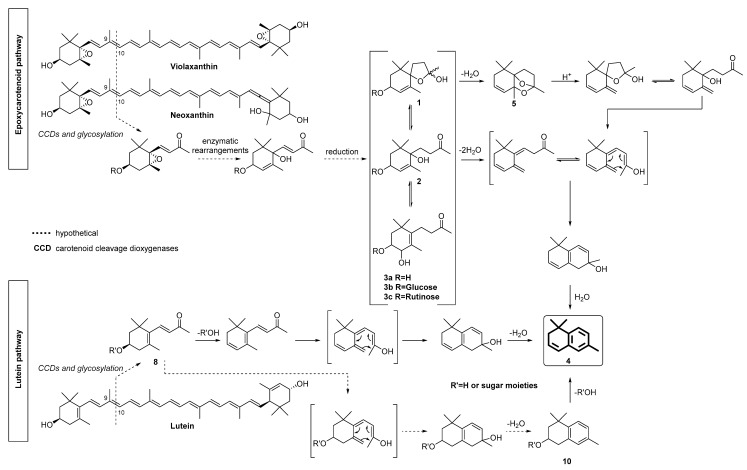
Postulated formation pathways of TDN formation starting from epoxy carotenoids (violaxanthin, neoxanthin) and lutein [18,19].

In the year 1990, Winterhalter and coworkers analyzed a Riesling wine extract with the all-liquid chromatographic technique of droplet countercurrent chromatography (DCCC). The DCCC-fractions were hydrolyzed by simultaneous distillation/extraction at pH 3.2 and the generated TDN was monitored by GC analysis. It could be shown that TDN is liberated from at least three precursor fractions exhibiting different polarities [8]. During subsequent studies, including enzymatic hydrolyses of the non-volatile glycosidic progenitors, 3,6-dihydroxy-7,8-dihydro-α-ionone **2,** which is in chemical equilibrium with the diastereomeric hemiacetals 2,6,10,10-tetramethyl-1-oxaspiro[4.5]ldec-6-ene-2,8-diols **1** and the allylic rearranged 3,4-dihydroxyketone **3a**, could be identified as TDN-generating aglycones (Figure 1) [8,14,15]. The hydrolysis of synthetic hemiacetals **1** mainly led, besides TDN, to a formation of 2,2,6,8-tetramethyl-7,11-dioxatricyclo[6.2.1.0]undec-4-en (Riesling acetal, **5**), which is also known to act as a progenitor of TDN **4** [16]. Based on biosynthetic considerations, it has been suggested that these highly oxygenated TDN progenitors are formed through the degradation of the epoxycarotenoids neoxanthin and violaxanthin [17,18].

In addition to compounds **1–3a**, at least one further less polar precursor with still unknown structural features exists, which, upon thermal treatment, generates only TDN and no Riesling acetal at all [9,17,19]. Attempts to isolate the intact TDN progenitors are rare and structural characterization is so far only based on the enzymatically liberated aglycones [14] or, more recently, on LC-TOF-MS analyses of the glycosides [20]. The reasons are obvious: the trace amounts, as well as the reactivity of these acid-labile progenitors, require very gentle isolation techniques that work on a preparative scale and hence enable an enrichment of the intact target compounds for subsequent structural characterization using NMR techniques. In particular, the low concentrations of the individual precursors are a challenge, which means that, for a successful isolation protocol, considerable amounts of Riesling wine (up to 100 L) have be worked up by the adsorptive enrichment of the glycosidic fraction on Amberlite XAD-2 adsorber resin. From the complex mixture of glycosidic components thus obtained, activity-controlled fractionation is able to localize those fractions that exhibit TDN-generating activities. However, this requires the fractionation of several grams of glycosidic extract, which is difficult to achieve with conventional separation techniques. A most suitable method for such gentle preparative fractionations is the all-liquid chromatographic technique of countercurrent chromatography, which has been used as the first separation step in this study. With countercurrent chromatography, the sample load can amount to up to several grams, depending on the type of chromatograph used.

In this study, we present the results of a first successful attempt to isolate intact TDN-generating glycoconjugates from a Riesling wine extract using an activity-guided isolation protocol by means of GC-MS/MS and high-performance countercurrent chromatography (HPCCC) as an initial fractionation step.

## 2. Results and Discussion

Climatic changes are increasingly influencing wine growing throughout the world. Cool climate wine regions that are famous for certain varieties with a distinct character (autochthonous grape varieties) are suffering from the temperature increase and, in Germany, mainly white wine producers are facing new challenges due to global warming [21]. This is especially true for Riesling, Germany’s premier grape variety not only in terms of area (23.3% of all plantings in 2018) but also with regard to quality [6]. Riesling grapes produce elegant wines of rich character with an incomparable aroma and taste. Unfortunately, under warmer climatic conditions, Riesling is prone to develop a so-called “petrol or kerosene” off-flavor that is caused by 1,1,6-trimethyl-1,2-dihydronaphthalene (TDN) **4** [1]. The general pathways of TDN formation is shown in Figure 1 (upper part), but there are still some gaps in our current knowledge, which prompted us to look deeper into TDN generation by clarifying the structures of individual TDN progenitors that exist in wine. First attempts in this regard have been made in the early 1990s [8,22,23]. Through the application of the all-liquid chromatographic technique of droplet countercurrent chromatography (DCCC), the existence of multiple precursors of the target compound TDN could be demonstrated for the first time. However, the complexity of the extract and the small amount of individual progenitors present in wine hampered a complete structural characterization of the genuine TDN-generating glycoconjugates so far.

### 2.1. Target-Guided Isolation of TDN Progenitors by High Performance Countercurrent Chromatography (HPCCC)

**Figure 2:**
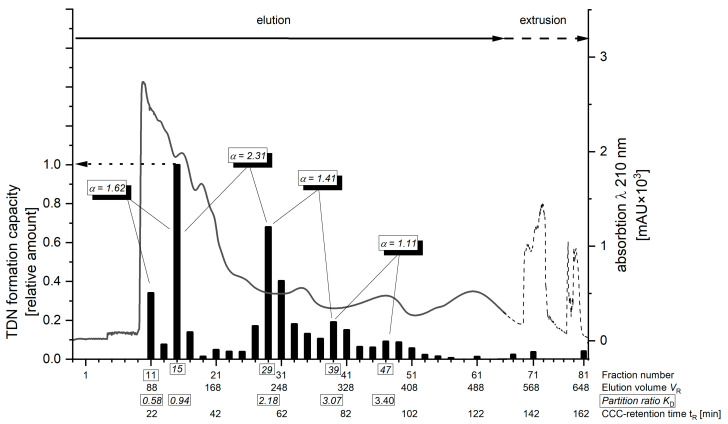
UV-chromatogram (λ 210 nm) of the HPCCC separation (right axis, solid and dotted line) and screening of TDN generating fractions by HS-GC-MS/MS (left axis, bar chart). α-values of CCC separation are given for TDN precursors. These values refer to the specific positions in the chromatogram that showed higher TDN formation and thus represent the separation of the structurally different precursors. The values are sufficient if they are greater than 1.5. (“calculations of countercurrent chromatographic separation parameters” are given in supplementary material).

In the last decades, considerable improvements have been made with regard to CCC instrumentation. Compared to the earlier used hydrostatic techniques, such as, e.g., DCCC, novel hydrodynamic separation systems of high-speed (HSCCC) or high-performance countercurrent chromatography (HPCCC) use higher *g*-forces (up to 240× *g*) which, inter alia, allow for increased flow rates. As a result, the separation time is reduced (in the present case, to approximately 150 min compared to 48 h for DCCC) and the improved separation processes significantly increase the separation efficiency. To avoid the traditional solvent systems for DCCC, which typically contain chloroform as the bottom layer, several more environmentally friendly solvent systems were sought. In order to evaluate suitable solvent systems, the distribution of the compounds in the glycosidic crude extract (E) of Riesling wine in the two layers of shake-flask experiments [24] was monitored by thin-layer chromatography. The distribution in the upper phase (UP) and lower phase (LP) was analyzed after visualization of the spots using anisaldehyde–sulfuric-acid–glacial-acetic-acid universal reagent (cf. Supplementary Table S1, Figure S1) [25]. Based on this screening, the solvent system composed of TBME/n-butanol/acetonitrile/water (2/2/1/5; *v*/*v*/*v*/*v*) was used in the head-to-tail mode for the HPCCC separation of the glycosidic extract from 100 L Riesling wine, yielding a total of 85 factions. After each CCC separation, the screening of every second separated fractions for TDN precursors was performed by the acid-catalyzed hydrolysis of the fractions at pH 3.0 for 2 h at 100 °C followed by a rapid TDN screening employing headspace (HS)-GC-MS/MS analysis. For HS-GC-MS/MS analyses, the selected reaction monitoring (SRM) mode was applied and a deuterated analogue of the target compound ([^2^H_6_]-TDN) was used as an internal standard for quantitation. The result of this screening is shown in Figure 2. Equations for the calculation of the parameters for countercurrent chromatographic separation (i.e., elution volume, partition ratio, etc.) can be found in the Supplementary Information (Equations (1)–(6)).

The result of the HPCCC fractionation was generally comparable to the separation previously achieved using DCCC [8]. Fractions 9–10 were not used for further separation as these fractions are still very complex and not part of the HS-GC/MS/MS screening experiment (cf. Figure 2 and Supplementary Figure S2). Three major TDN-producing regions were evident: the HPCCC polar fractions 11–18, the intermediate polar fractions 27–36 and the least polar fractions >36. The small amount of TDN formed during extrusion (fractions > 65) was not investigated further.

### 2.2. Isolation and Structure Elucidation of 3,4-Dihydroxy-7,8-dihydro-β-ionone 3-0-β-D-glucopyranoside 3b and 3,4-Dihydroxy-7,8-dihydro-β-ionone 3-0-rutinoside 3c from the Polar HPCCC Fractions 11–18

For the isolation of rutinoside **3c**, further purification steps of combined HPCCC fractions 11–13 (~*K*_D_ = 0.61; Figure 2) were required, consisting of gel permeation chromatography on Sephadex LH-20 followed by a work-up on a silica gel column and the final purification on a RP-18 semi-preparative column. After each separation step, the fractions were screened by HS-GC-MS/MS as described above. The NMR spectral data of pure **3c** confirmed the structure of 3,4-dihydroxy-7,8-dihydro-β-ionone 3-0-rutinoside.

**Spectral data of 3,4-dihydroxy-7,8-dihydro-β-ionone 3-0-rutinoside 3b. ^1^H-NMR** (600 MHz, CD_3_OD, δ ppm): 1.05 (s, 3H, H-12), 1.07 (s, 3H, H-11), 1.53–1.57 (m, H, H-2a), 1.75 (s, 3H, H-13), 1.87 (t, H, *J* = 12.9 Hz, H-2b), 2.14 (s, 3H, H-10), 2.18–2.33 (m, 2H, H-7), 2.58 (t, 2H, 8,3 Hz, H-8), 3.83–3.89 (m, H, H-3), 3.95–3.99 (m, H, H-4), 3.18–3.23 (m, H, H-2′), 3.27–3.33 (m, H, H-4′), 3.34–3.38 (m, H, H-3′), 3.39–3.43 (m, H, H-5′), 3.56–3.60 (m, H, 6a’), 3.95–3.99 (m, H, 6b’), 4.44 (d, H, *J* = 7.9 Hz, H-1′), 1.25 (d, 3H, *J* = 6.1 Hz, H-6″), 3.34–3.38 (m, H, H-4″), 3.62–3.66 (m, H, H-2″), 3.62–3.66 (m, H, H-5″), 3.81 (dd, H, *J* = 1.7/3.5 Hz, H-3″), 4.71 (d, H, *J* = 1.7 Hz, H-1″). **^13^C-NMR** (150.9 MHz, CD_3_OD, δ ppm)**:** 39.0 (C_q_, C-1), 40.4 (CH_2_, C-2), 76.5 (CH, C-3), 70.7 (CH, C-4), 128.2 (C_q_, C-5), 143.3 (C_q_, C-6), 23.1 (CH_2_, C-7). 44.3 (CH_2_, C-8), 211.4 (C_q_, C-9), 29.7 (CH_3_, C-10), 27.7 (CH_3_, C-11), 29.6 (CH_3_, C-12), 18.3 (CH_3_, C-13), 103.2 (CH, C-1′), 75.3 (CH, C-2′), 77.9 (CH, C-3′), 71.6 (CH, C-4′), 76.8 (CH, C5′), 68.0 (CH_2_, C-6′), 102.1 (CH, C-1″), 72.4 (CH, C2″), 72.2 (CH, C3″), 74,1 (CH, C-4″), 69,8 (CH, C-5″), 18.2 (CH_3_, C-6″).

Additionally, the same TDN-precursor was also isolated as a monoglucoside from the second-TDN-generation region, i.e., HPCCC-fractions 14–16 (~*K*_D_ = 0.94). In this case, after gel chromatography, a purification on an analytical C18-column was necessary to obtain pure **3b**. The HPLC-ESI-MS data already pointed out that the precursor was linked to a simple hexose due to a neutral loss of 162 Da, and the NMR spectral data confirmed the structure as 3,4-dihydroxy-7,8-dihydro-β-ionone 3-0-β-D-glucopyranoside **3b**, which has earlier been isolated from Riesling leaves [13].

**Spectral data of 3,4-dihydroxy-7,8-dihydro-β-ionone 3-0-β-D-glucopyranoside 3c. ^1^H-NMR** (600 MHz, CD_3_OD, δ ppm): 1.05 (s, H, H-12), 1.06 (s, 3H, H-11), 1.55 (ddd, H, *J* = 1.5/ 3.6/ 12.4 Hz, H-2a), 1.75 (s, H, H-13), 1.85 (t, H, *J* = 12.7 Hz, H-2b), 2.14 (s, 3H, H-10), 2.20–2.27 (m, H, H-7a), 2.27–2.22 (m, H, H-7b), 2.57 (t(br), H, *J* = 8.3 Hz, H-8), 3.93 (dt, H, *J* = 3.7/ 12.9 Hz, H-3), 3.95–4.03 (m, H, H-3), 3.21 (dd, H, *J* = 7.8/ 9.2 Hz, H-2′), 3.28–3.30 (m, 2H, H-4′/H-5′), 3.36.-3.39 (m, H, H-3′),3.65–3.68 (m, H, 6a′), 3.84–3.87 (m, H, 6b’), 4.48 (d, H, *J* = 7.8 Hz, H-1′). **^13^C-NMR** (150.9 MHz, CD_3_OD, δ ppm)**:** 18.4 (CH_3_, C-13), 23.1 (CH_2_, C-7), 27.6 (CH_3_, C-11), 29.6 (CH_3_, C-12), 29.7 (CH_3_, C-10), 38.9 (C_q_, C-1), 40.1 (CH_2_, C-2), 44.3 (CH_2_, C-8), 76.1 (CH, C-3), 70.4 (CH, C-4), 128.1 (C_q_, C-5), 143.3 (C_q_, C-6), 211.1 (C_q_, C-9), 62.7 (CH_2_, C-6′), 71.6 (CH, C-4′),75.4 (CH, C-2′), 78.0 (CH, C-3′), 78.1 (CH, C5′), 102.7 (CH, C-1′).

The signals in the ^1^H spectra at δ = ~1 ppm are typical for the geminal CH_3_ groups on the cyclohexane ring. In the ^13^C spectrum, 13 carbon signals appear in addition to the sugar signals. The signals of the double bond at δ = 128 and 143 ppm and of the quaternary C atom at δ = 211 ppm, which is identified as a keto group, are particularly noticeable. The anomeric C atom is identified as a beta anomer by the doublet δ = ~4.4 ppm, with a coupling constant of *J* = 7.8 Hz. Due to the linkage to the rhamnose, the diastereotopic protons of C6’ of the diglycoside are paramagnetically shifted compared to a monoglycosidic compound [26]. The obtained data for diglycoside are comparable to a rutinoside of a monoterpene derivative published [27]. The signals for carbon atom C1″ of α-L-rhamnose with a chemical shift of δ = 4.71 ppm and a coupling constant of *J* = 1.7 ppm, as well as for the methyl group at C6″ with δ = 1.25 ppm and *J* = 6.1 Hz, are identical to the published data of the rutinose moiety of a stilbene derivative [28].

The results of the HSQC and HMBC experiments for **3b** and **3c**, as well as mass spectrometric data, are given in Supplementary Table S2–S4.

In the case of the less polar HPCCC fractions 27–35 (~*K*_D_ = 2.10), the isolation of intact precursors failed because of the complexity of the fractions. Hence, enzymatic hydrolysis was conducted in order to liberate TDN-generating aglycones for subsequent GC-MS analysis. Importantly, the known lutein degradation product 3-hydroxy-ß-ionone **8** could be detected after enzymatic hydrolysis, indication that further TDN-generating precursors related to the major grape carotenoid lutein also exist in a glycosidically bound form in wine. Together with ketone **8**, the structurally related 3-hydroxy-TDN **10** was detected as aglycone in trace amounts. This compound has earlier been identified as a TDN precursor in a glycosidic extract from passion fruit [10]. The presence of **8** and **10** indicates that TDN formation is not only restricted to the epoxycarotenoids neoxanthin and violaxanthin. The grape carotenoid lutein is most likely another source of TDN-generating intermediates, although it has not yet been clarified why only such small amounts of the metabolites **8** and **10** can be detected, since lutein is one of the main carotenoids in Riesling grapes. In further less polar TDN-generation fractions (36–44 and 45–50), glycosidic progenitors were absent. It can be hypothesized that the formation of TDN in this region is most likely due to non-conjugated components, with the so-called Riesling acetal **5** being a likely candidate.

**Figure 3:**
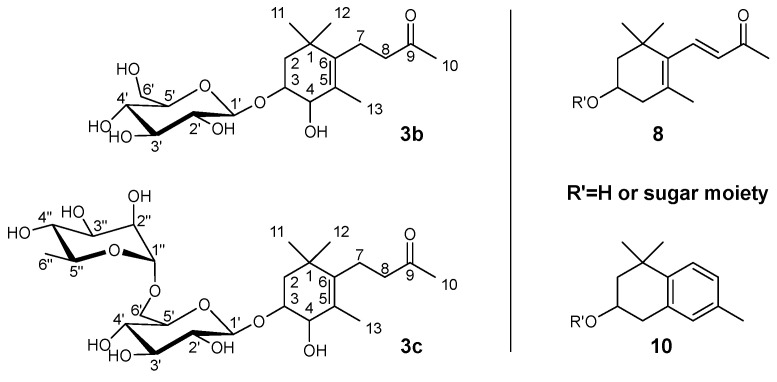
Structures of the newly isolated glycosylated TDN precursors **3b** and **3c**, as well as the enzymatically liberated aglycones 3-hydroxy-ß-ionone **8** and 3-hydroxy-TDN **10**.

An extended pathway for TDN formation in Riesling wine that also includes lutein as an initial carotenoid precursor is shown in Figure 1. The structures of the so-far-isolated TDN-generating glycosides **3b/c**, as well as the additionally liberated TDN-generating aglycones **8** and **10**, are shown in Figure 3.

## 3. Materials and Methods

### 3.1. Chemicals and General Experimental Conditions

All chemicals and solvents were obtained commercially with appropriate quality and used without further purification. Formic acid, HPLC-grade acetonitrile (ACN), tert.-butyl methyl ether (TBME), 1-butanol, methanol were obtained from Merck (Darmstadt, Germany). *n*-Pentane, *n*-hexane, dichloromethane and ethyl acetate were of analytical grade (VWR Chemicals, Fontenay-sous-Bois, France). For all experiments, deionized water (NANOpure^®^, Werner, Leverkusen, Germany) was used.

Analytical standards were synthesized as described earlier [5,29]. Synthesized reference substances were purified by means of silica gel chromatography and the individual purity was checked by GC analysis and spectroscopic methods, ensuring sufficient purity for quantitative analyses. For column and flash chromatography, silica gel 0.04–0.063 mm (230–400 mesh ASTM, Merck; Darmstadt, Germany) was used. Thin layer chromatography (TLC) was performed on silica gel 60 F_254_ plates (Merck, Darmstadt, Germany). Citric acid and disodium hydrogen phosphate dihydrate for the preparation of McIlvaine buffer were of analytical grade and were obtained from Merck (Darmstadt, Germany).

### 3.2. Isolation of Glycosidically Bound TDN Precursors

**Figure 4:**
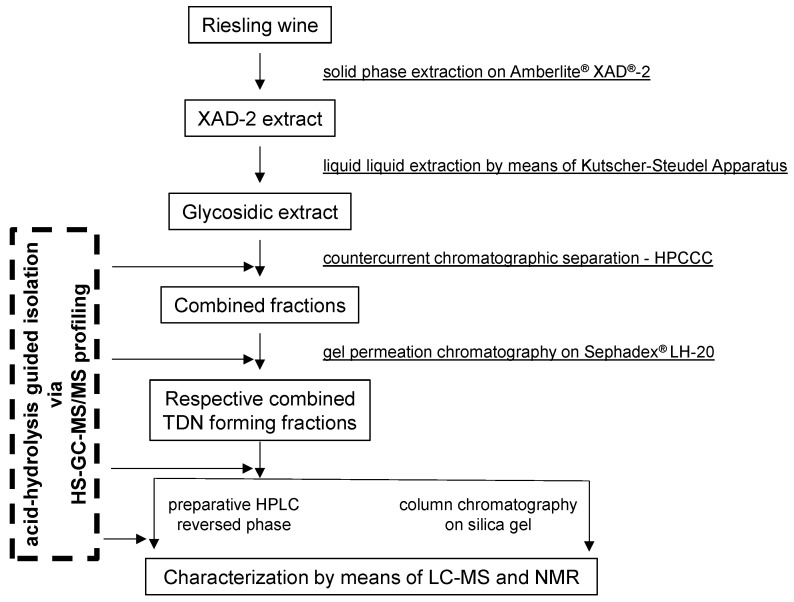
Overview of isolation steps of TDN-generating precursors.

A commercial Riesling wine (100 L Pfalz Riesling QbA from the Moselland eG Winzergenossenschaft, Germany, vintage 2012) was used in this study. The extraction was conducted according to previous studies [30,31], and the different isolation steps are summarized in Figure 4. The Riesling wine (batches of 10 L) was first diluted with water (1/1, *v*/*v*) and passed through a column (80 cm × 5 cm) of Amberlite^®^ XAD^®^-2 resin. Afterwards, the column was thoroughly rinsed with water (6 L) before the precursor fraction was eluted with methanol (3 L). The methanolic fractions were combined, carefully concentrated in vacuo (30 °C) and lyophilized. The redissolved batches of this residue (aprox. 5 g in 200 mL water) were extracted by liquid–liquid extraction (Kutscher–Steudel perforator) with *n*-pentane/dichloromethane (*v*/*v*; 2/1; 500 mL) and lyophilized. Aliquots (0.5–1.0 g) of the lyophilized extract (23 g) were subjected to HPCCC fractionation and subsequent purifications by gel permeation chromatography (Sephadex LH-20), as well as semi-preparative and/or analytical RP-18 HPLC, respectively. TDN-generating fractions were monitored by acid-catalyzed hydrolysis of the non-volatile precursors at pH 3.0 and subsequent headspace (HS)-GC-MS/MS analysis after each purification step.

The most polar TDN-generating HPCCC-fractions (11–13) were further fractionated with gel permeation chromatography (Sephadex LH-20), silica gel column chromatography (dichloromethane/methanol; 75/25; *v*/*v*) and semi-preparative HPLC, and delivered compound **3c** as a white foamy solid (2.0 mg). In addition, the second TDN-generating region (polar HPCCC fractions 14–16) was further fractionated by gel permeation chromatography (Sephadex LH-20). After HS-GC-MS/MS screening of the GPC-separation, TDN-generating fractions were cleaned up on an analytical HPLC system and **3b** (1.2 mg) was obtained as foamy solid.

### 3.3. HPCCC Fractionation of the XAD-2 Extract from Riesling Wine

For the preparation of the HPCCC solvent system, TBME/*n*-butanol/acetonitrile/water (2/2/1/5; *v/v/v/v*) was mixed in a separation funnel until the phases were saturated with each other. After separation of the two layers, the HPCCC separation was carried out in head-to-tail modus, i.e., the upper, less dense layer was used as stationary phase [24]. After starting the rotation of the HPCCC system at 1.600 rpm, the more dense aqueous layer was pumped into the system as mobile phase at a flow rate of 4 mL/min. Aliquots of 1 g of the glycosidic XAD-2 extract were dissolved in mobile phase and injected via a sample loop (0.5–1 g extract/5 mL). Fractions were collected every 2 min. After 130 min, the system was set to extrusion mode [32], i.e., the rotation was reduced to 200 rpm while the stationary phase was pumped into the system at a flow rate of 8 mL/min. During extrusion, fractions were collected every min. The HPCCC was a Spectrum model from Dynamic Extractions Ltd. (Gwent, UK) with a column volume of 125.5 mL and a column i.d. of 1.6 mm. The HPCCC was equipped with a K-501 pump and a Wellchrom K-2600 detector (both from Knauer, Berlin, Germany), as well as a Superfrac fraction collector (Pharmacia, Uppsala, Sweden) and an RC-6 cooling system (Lauda, Königshofen, Germany).

### 3.4. Gel Permeation Chromatography (GPC)

TDN-generating fractions (160 mg to 1000 mg) from the initial HPCCC fractionation were concentrated, combined in groups, lyophilized and further purified with a Sephadex LH-20 column (1.5 cm × 1.5 m) using the mixture of methanol/ethyl acetate (1/1; *v/v*) as eluting solvent. The flow rate was approximately 0.5 mL/min to 0.7 mL/min and the fraction size was set to 10 min using a Superfrac fraction collector (Pharmacia, Uppsala, Sweden).

### 3.5. Further Purification by Semi-Preparative and Analytical HPLC

As next step, semi-preparative purifications of the TDN-generating GPC-fractions on an HPLC system from Knauer (Berlin, Germany) with a Smartline Pump 1000 combined with a degasser and gradient system (Smartline Manager 5000) and a WellChrom UV detector K-2600 at a wavelength of 210 nm were carried out. HPLC separations were performed on a Pursuit XRs C18 column (250 mm × 10 mm, 5 μm, Agilent Technologies) with water (A) and acetonitrile (B) as mobile phase at a flow rate of 2.5 mL/min. Conditions: 0 min (10% B), 90 min (20% B), 95 min (10% B), 100 min (10% B). For further analytical purifications, an HPLC system (LC1220 Series from Agilent Technologies, Waldbronn, Germany) equipped with a Luna Phenyl-Hexyl column (250 mm × 4.6 mm, 3 μm, Phenomenex, Aschaffenburg, Germany) with a Grad Pump (G4288C) and a VWD detector (G4288C) was used. Separations were carried out with water (A) and acetonitrile (B) at a flow rate of 0.5 mL/min under the following conditions: 0 min (10% B), 90 min (20% B), 95 min (10% B), 105 min (50% B), 110 min (10% B), 125 min (10% B).

### 3.6. Off-Line Headspace (HS)-GC-MS/MS Screening of TDN-Generating Fractions

The detection of TDN-generating fractions was conducted by a countercurrent chromatography novel fast screening method using HS-GC-MS/MS analysis. First, the stability of labelled TDN standard was investigated. Internal standard was added to McIlvaine buffer (citrate-phosphate-buffer, pH adjusted to 3.0; pH-value of the hydrolysis experiments) and heated to 100 °C for 2 h. HS-GC-MS/MS analysis under same conditions as samples was carried out to check if any exchange had occurred (Supplementary information Figure S3).

To a total of 10 to 100 µL of each HPCCC fraction (gently dried under nitrogen stream), McIlvaine buffer (pH 3.0) and deuterated standard were added to maintain concentration 50 µg/L d6-TDN. The GC vials were capped and samples were hydrolyzed at 100 °C for 2 h. After hydrolysis, the samples were cooled to room temperature. The GC analysis was performed with a TRACE 1300 gas chromatograph equipped with a TriPlusRSH autosampler (both Thermo Fisher Scientific, Waltham, Massachusetts, USA). Headspace injections of the samples (2 µL, post injection delay:15 s) without further incubation and agitation were performed in a programmable temperature vaporizing (PTV) inlet at 240 °C in splitless mode, and purged after 2 min. This injector was equipped with a 2.0 mm (i.d.) metal liner (ThermoFisher Scientific). A polar deactivated guard column (2.5 m × 0.53 mm i.d.) from Restek (Bellefonte, PA, USA) was coupled via a “T” connector (ThermoFisher Scientific) to a VF-WAXMS analytical column (30 m × 0.25 mm i.d. × 0.25 μm d_f_) from Agilent Technologies (Waldbronn, Germany), and a backflush was set to 90 sec. Carrier gas was helium with a constant flow rate of 1.6 mL/min. The oven temperature program started at 50 °C and increased to 240 °C at 80 °C/min, and was held isothermally for 1 min. The mass spectrometric (MS) detection was carried out with a TSQ Duo triple quadrupole mass spectrometer (ThermoFisher Scientific). Electron impact ionization (EI) was applied (70 eV). Temperatures for the MS transfer line and ion source were set to 250 °C. Argon (purity ≥ 99.999%) was used as collision gas (4.2 bar). Mass resolution was set to 1 amu for Q1 and Q3 (cycle time 200 ms). Mass transitions (SRMs; *m/z*) and collision energies are listed hereafter (quantifier ions are underlined): 157.1 → 142.1 (14 V), 157.1 → 115.1 (38 V), 172.1 → 157.1 (8 V) for TDN and 163.2 → 148.2 (10 V), 145.1 → 144.1 (15 V), 178.2 → 163.2 (10 V) for TDN-d_6_. Xcalibur software (version 3.0.63) was used for instrument control and data acquisition (ThermoFisherScientific).

### 3.7. HPLC-ESI-MS/MS-Analysis

For HPLC-ESI-MS/MS determinations, a HPLC System from Agilent Technologies (Waldbronn, Germany) consisting of a Bin Pump (G1312A, 1100 Series) and an ALS SL (G1329, 1200 Series) connected to HCT-ultra PTM Discovery MS System from Bruker Daltonics (Bremen, Germany) was used. The ESI-MS/MS ion-trap system was operated alternating in positive and negative mode, the dry gas was nitrogen with a flow rate of 11.0 L/min, temperature was set at 330 °C, capillary voltage was −3500 V and end plate offset was −500 V. The nebulizer was set to 60.0 psi and the target mass was *m*/*z* 500, with a scan range from *m/z* 100 to 1000. The separation was conducted on an Aqua C18 column (150 × 2.0 mm, 3 μ) from Phenomenex (Aschaffenburg, Germany) at a flow rate of 0.2 mL/min. Mobile phase consisted of water (A) and acetonitrile (B), including 0.05% formic acid in both solvents. HPLC conditions were as follows: 0 min (1% B), 20 min (20% B), 35 min (50% B), 37 min (100% B), 42 min (100% B), 45 min (1% B), 60 min (1% B).

### 3.8. Enzymatic Hydrolysis of TDN-Generating HPCCC Fractions

For enzymatic hydrolysis, four consecutive fractions of the HPCCC separation were combined in each case and lyophilized. Approximately 10 mg of each of these combined fractions were dissolved in citrate–phosphate buffer [33,34] at a defined pH value of 5.0. Prior to enzymatic hydrolysis, the fractions were extracted with 100 µL *n*-hexane/dichloromethane (2/1, *v*/*v*) to ensure the absence of volatile compounds. A total of 500 µL of Rapidase AR2000 enzyme suspension was then added and hydrolysis was conducted for 21 h at 37 °C. The enzyme suspension was prepared by adding 140 mg Rapidase AR2000 (DSM Food Specialties, Netherlands) to 1 mL citrate–phosphate buffer (pH-value of 5.0). After hydrolysis, the solution was cooled to room temperature and extracted three times with 500 µL of a mixture of n-hexane/dichloromethane (2/1; *v*/*v*). The combined organic phases were dried over sodium sulfate, concentrated to 150 µL and analyzed by GC-MS (TIC: total ion current). The same GC-system as for headspace determinations was used and sample volumes of 1 µL were injected in splitless mode and purged after 2 min. The oven temperature program started at 50 °C, increased to 240 °C at 10 °C/min and was held isothermally for 20 min. Carrier gas was helium with a constant flow of 1.2 mL/min. Backflush was set to 39.5 min.

### 3.9. Nuclear Magnetic Resonance (NMR) Spectroscopy

NMR experiments were performed on a Bruker Avance II 600 spectrometer (Bruker, Rheinstetten, Germany). Samples were recorded at room temperature in methanol-d4 (>99.96% D, Deutero GmbH, Kastellaun, Germany) and referenced to tetramethylsilane (TMS, δ = 0 ppm; Sigma-Aldrich, Deisenhofen, Germany) as internal standard. Chemical shifts δ were reported in parts per million (ppm), coupling constants *J* in Hertz (Hz).

## 4. Conclusions

In this first successful attempt to isolate intact TDN-forming glycoconjugates from a Riesling wine extract, we presented the results of a novel activity-guided isolation protocol using an HS-GC-MS/MS assay employing a deuterated analogue of the target compound as well as high-performance countercurrent chromatography (HPCCC) as a first fractionation step. The elucidated structures of the isolated mono- and di-glycosylated TDN precursors provide a reliable starting point for detailed studies in future on the formation pathway of TDN. On the other hand, the enzymatically liberated aglycones of further precursors, i.e., 3-hydroxy-β-ionone and 3-hydroxy-TDN, allow us to propose an extended pathway for TDN formation in Riesling wine that also includes the major grape carotenoid lutein as initial precursor.

## Data Availability

The data that support the findings of this study are available from the corresponding authors upon reasonable request.

## Supplementary Materials

The following supporting information can be downloaded at: https://www.mdpi.com/article/10.3390/molecules27175378/s1, Table S1. Investigated CCC solvent systems for Amberlite^®^ XAD^®^-2 extract of Riesling wine; Figure S1. TLC analysis of the suitable solvent system prediction experiments; distributions in the phase layers of shake-flask experiments upper-phase-UP–lower-phase-LP, crude extract E. Normal phase silica gel TLC plates were developed with ethyl acetate/methanol/water 75:25:5 (*v*/*v*/*v*). Visualization was performed using anisaldehyde–sulfuric-acid–glacial-acetic-acid universal reagent; Figure S2. TLC analysis of the CCC fractions; (a) fractions 9–44 (b) fractions 45–80. Normal phase silica gel TLC plates were developed with ethyl acetate/methanol/water 7:3:0.5 (*v*/*v*/*v*). Visualization was performed using anisaldehyde–sulfuric-acid–glacial-acetic-acid universal reagent; Figure S3. Stability of deuterated TDN under hydrolysis conditions; Figure S4. Structure-relevant long-range HC-correlation signals observed in the HMBC of 3b; Figure S5. Structure-relevant long-range HC-correlation signals observed in the HMBC of 3c; Equations (1)–(7); Table S2. ESI-MS/MS data of isolated compounds (3b,c); Table S3. NMR data of 3,4-dihydroxy-7,8-dihydro-β-ionone 3-O-β-D-glucopyranoside (3b); Table S4. NMR data of 3,4-dihydroxy-7,8-dihydro-β-ionone 3-O-rutinoside (3c).

## Sample Availability

Samples of the compounds are not available from the authors.
