# Supplementary material for: Target-Guided Isolation of Progenitors of 1,1,6-Trimethyl-1,2-dihydronaphthalene (TDN) from Riesling Wine by High-Performance Countercurrent Chromatography
Source: Molecules. 2022 Aug 23;27(17):5378. doi: 10.3390/molecules27175378 (PMC9458034; doi:10.3390/molecules27175378)
Supplement: Supplementary file 1 [file molecules-27-05378-s001.zip › molecules-1844681-supplementary.pdf]

Supplementary Material

Target-Guided Isolation of Progenitors of 1,1,6-Trimethyl-1,2-dihydronaphthalene (TDN) from Riesling Wine by High-Performance Countercurrent Chromatography †

Recep Gök [1,]*, Pia Selhorst [1], Mats Kiene [1], Theresa Noske [1], Michael Ziegler [2], Ulrich Fischer [2] and Peter Winterhalter [1,]*

[1] Institute of Food Chemistry, Technische Universität Braunschweig, Schleinitzstrasse 20, 38106 Braunschweig, Germany; p.selhorst@tu-braunschweig.de (P.S.); m.kiene@tu-braunschweig.de (M.K.); t.noske@outlook.com (T.N.)
[2] Institute for Viticulture and Oenology, Dienstleistungszentrum Ländlicher Raum (DLR) Rheinpfalz, 67435 Neustadt an der Weinstraße, Germany; michael.ziegler@dlr.rlp.de (M.Z.); ulrich.fischer@dlr.rlp.de (U.F.)

* Correspondence: r.goek@tu-braunschweig.de (R.G.); p.winterhalter@tu-braunschweig.de (P.W.)

† In memoriam of Dr. phil. nat. habil. Hans-Georg Schmarr (1961–2019).

Solvent system prediction for countercurrent chromatograpy

Table S1. Investigated CCC solvent systems for Amberlite® XAD®-2 extract of Riesling wine [Ito, Y. 2005]

#	Solvent system	Composition (*v/v/v/v*)	Remarks
1	*n*-Hexane/Ethyl acetate/Methanol/Water	3:5:3:5	
2	*n*-Hexane/Ethyl acetate/Methanol/Water	5:5:5:5	
3	*n*-Hexane/Ethyl acetate/Methanol/Water	7:3:5:5	
4	*n*-Hexane/Ethyl acetate/Methanol/Water	1:5:1:5	
5	*n*-Hexane/Ethyl acetate/Methanol/Water	0:5:0:5	
6	Chloroform/Methanol/Water	7:13:8	Original solvent system
7	Dichlormethane/Methanol/Water	7:13:8	
8	*n*-Butanol/Water	1:1	Promising
9	*n*-Hexane/Ethyl acetate/Methanol/Water	7:3:7:3	
10	*tert*-Butyl methyl ether/Acetonitrile/Water	6:3:8	Promising
11	*tert*-Butyl methyl ether/Water	1:1	
12	*tert*-Butyl methyl ether/*n*-Butanol/Acetonitrile/Water	2:2:1:5	Selected solvent system

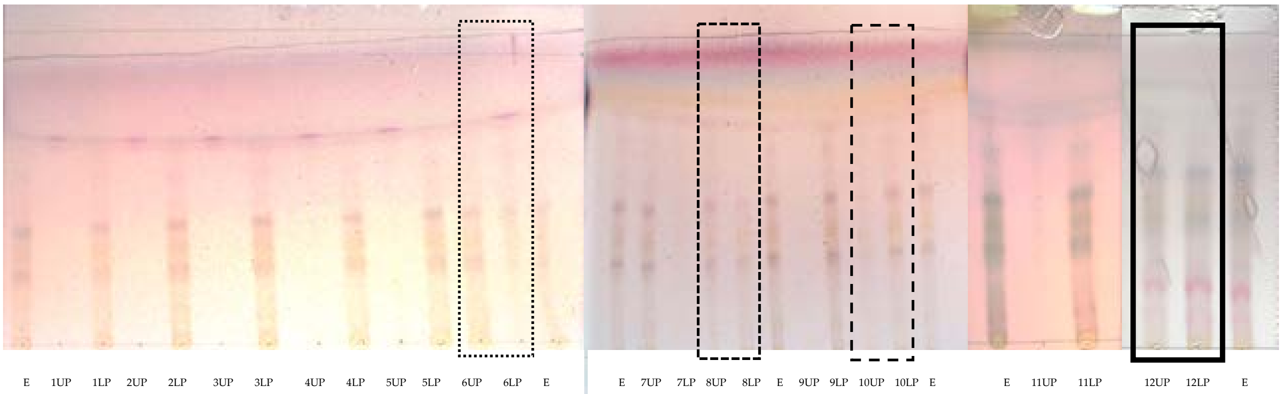

Figure S1. TLC analysis of the suitable solvent system prediction experiments; distributions in the phase layers of shakeflask experiments upper phase UP – lower phase LP, crude extract E [Ito, Y. 2005]. Normal phase silica gel TLC plates were developed with Ethyl acetate/Methanol/Water 75:25:5 (*v*/*v*/*v*). Visualization was done using anisaldehyde–sulfuric acid–glacial acetic acid universal reagent [Stahl, E. and Kaltenbach, U. 1961].

TLC screening of CCC fractions (exemplary)

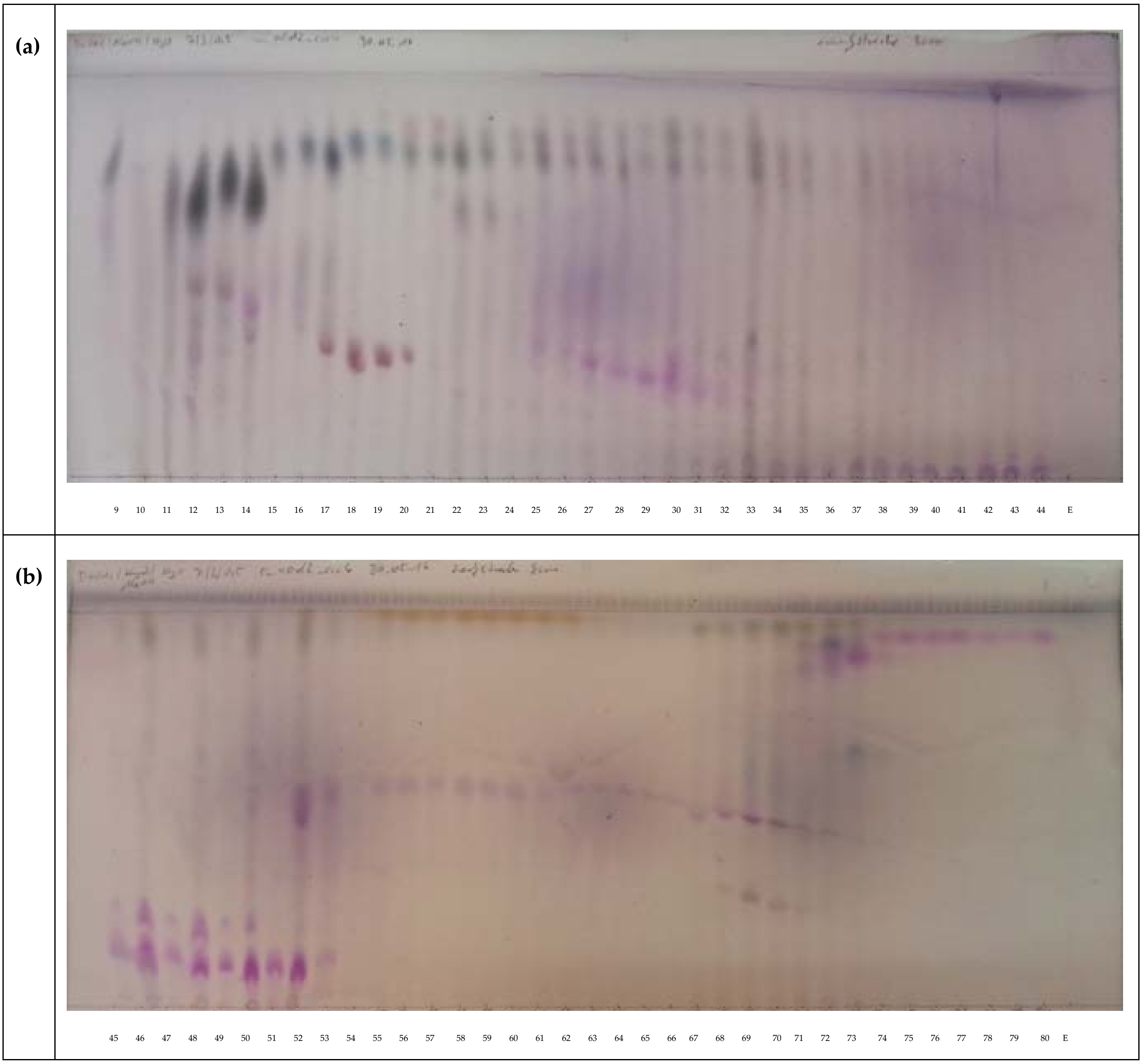

Figure S2. TLC analysis of the CCC fractions; a) fractions 9 – 44 b) fractions 45 – 80. Normal phase silica gel TLC plates were developed with Ethyl acetate/Methanol/Water 7:3:0.5 (*v*/*v*/*v*). Visualization was done using anisaldehyde–sulfuric acid–glacial acetic acid universal reagent. [Stahl, E. and Kaltenbach, U. 1961]

Stability of deuterated TDN for HS-GC-MS/MS-Screening method for generated fractions

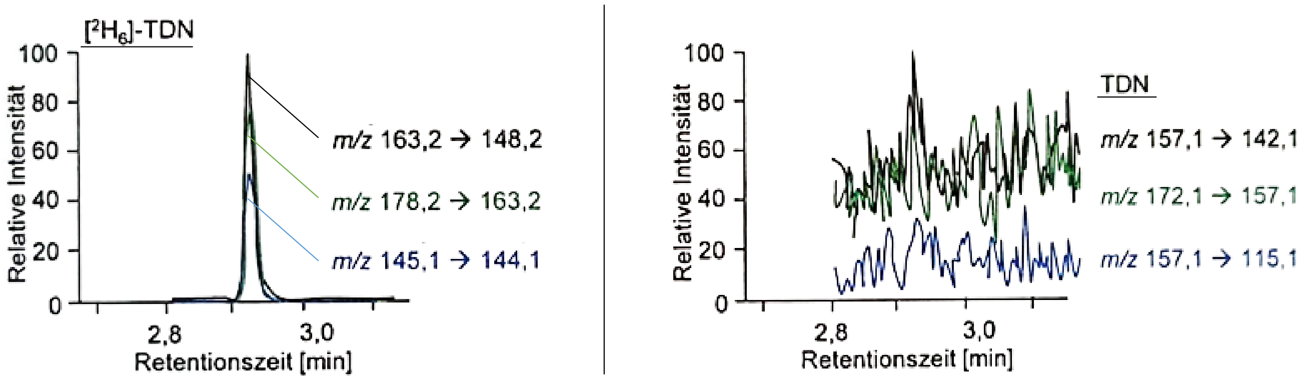

Figure S3. Stability of deuterated TDN under hydrolysis conditions.

Calculation of countercurrent chromatographic separation parameters

The chromatographic elution time was converted over *elution/retention volumes* V_R into their respective *partition ratio* values K_D (cf. equations 1-7). The experimental V_R values of screened fractions (HS-GC-MS/MS-Screening) were calculated with equation 1.

Retention volume V_R = elution time [min] x flow rate [mL/ min] *(1)*

The S_F values of the used solvent systems were determined by equation **2a-b** using V_C (125 mL), and V_M resulting in the S_F value measured at the hydrodynamic equilibrium.

$$V_S = (V_C - V_M) \quad \textbf{(2a)}$$
$$S_F = V_S / V_C \times 100\% \quad \textbf{(2b)}$$
$S_F = 67.2\%$

V_S : retained experimental stationary phase volume
V_C : CCC column volume/capacity (200 mL)
V_M : volume of mobile phase take up to the coil at equilibrium of CCC
V_{CM}: volume with change of elution to extrusion-mode (*switch volume*)
S_F : stationary phase retention
K_D: partition ratio

The determined S_F-value in the experiment is corrected by the *extra column volume* V_{Ext} (6 mL) of the connecting periphery tubing in the CCC set-up, using equations 3-5

Corrected $V_M = V_M - V_{ext}$ (3)
corr. V_M = 41 mL - 6 mL = 35 mL

Corrected $V_S = V_C$ - corrected V_M (4)
corr. V_S = 125 mL - 35 mL = 90 mL

corrected S_F = corrected V_S / V_C (5)
corr. S_F = 90 mL / 125 mL x 100% = 72%

A high S_F value directly correlate to a higher resolution and efficiency of the CCC-separation.

The solvent system specific partition ratio K_D values in the CCC run were calculated by the equations **(6)** (results cf. Figure 2). [Berthod et al. 2007]

During e*lution-mode:*

K_D = (V_R - corrected V_M) / corrected V_S **(6)**

The calculation of the **separation factor** α based on the K_D values:

$\alpha = K_{D2} / K_{D1}$ (with $K_{D2} > K_{D1}$) (7)

ESI-MS/MS and NMR Data of isolated compounds

Table S2. ESI-MS/MS data of isolated compounds (3b and 3c).

comp.	*Mw [g/mol]*	*ESI polarity*	*pseudo molecular ion*	*parent ion m/z*	*m/z from MS² ᵃ*
3b	*388*	+	*[M+NH₄]⁺*	406	371(91), 353(14), 209(42), 191(100), 173(45), 151(26), 133(38)
			[M-H₂O+H]⁺	371	209(26), 191(100), 173(98), 133(50)
		-	*[M-H]⁻*	387	225(16), 207(10), 189(7), 161(100)
			[M+CH₂O₂-H]⁻	433	388(16), 387(100), 224(16), 161(5)
**3c**	534	+	*[M-H₂O+H]⁺*	517	371(5), 319(6), 271(9), 261(8), 209(22), 192(7), 191(14), 190(5), 177(5), 174(7), 173(100), 171(5), 157(5)
		-	*[M+2H₂O-H]⁻*	569	534(18), 533(100), 389(3), 387(25), 163(4)

[a] Base peaks are underlined, brackets show the relative intensity

Table S3. NMR data of 3,4-dihydroxy-7,8-dihydro-β-ionone 3-*O*-β-D-glucopyranoside (3b).[a]

Position[b]	δ_C ppm (CH_n)	δ_H ppm; mult., *J* Hz[c]	HMBC[d]
1	38.9 (C_q)		
2	40.1 (CH_2)	1.55 (a); ddd *J*=1.5/3.6/12.4 Hz<br>1.85 (b); t *J*=12.7 Hz	(a) 1,3,4,6,11<br>(b) 1,3,4,11,12
3	76.1 (CH)	3.93; dt *J*=3.7/12.9 Hz	1,2,4,1′
4	70.4 (CH)	3.99; m	2,3,5,6,13
5	128.1 (C_q)		
6	143.3 (C_q)		
7	23.1 (CH_2)	2.20-2.33; m	1,5,6,8,9
8	44.3 (CH_2)	2.57; t(br) *J*=8.3 Hz	6,7,9
9	211.1 (C_q)		
10	29.7 (CH_3)	2.14; s	8,9
11	27.6 (CH_3)	1.06; s	1,2,6,12
12	29.6 (CH_3)	1.05; s	1,2,6,11
13	18,4 (CH_3)	1.75; s	4,5,6
1′	102.7 (CH)	4.48; d *J*=7.8 Hz	3,3′
2′	75.4 (CH)	3.21; dd *J*=7.8/9.2 Hz	1′,3′
3′	78.0 (CH)	3.36-3.39; m	1′,2′,4′,5′
4′	71.6 (CH)	3.28-3.30; m	4′,6′
5′	78.1 (CH)	3.28-3.30; m	1′,4′,6′
6′	62.7 (CH_2)	3.65-3.68 (a)<br>3.84-3.87 (b)	(a) 4′,5′<br>(b) 4′,5′

[a] Solvent: CD_3OD; tetramethylsilane (δ = 0.00 ppm) for ^1H, CD_3OD (δ = 49.00 ppm) for ^{13}C; ^1H observation frequency 600.1 MHz.
[b] For numbering of the carbon atoms, see the formula, assignment of C-H via HSQC data..
[c] For CH_2 groups with diastereotopic protons a and b indicate the deshielded and shielded nucleus, respectively.
[d] Entries in the column HMBC indicate ^1H nuclei showing long-range correlations ($^{2,3}J$) with the ^{13}C chemical shift in the second column.
n hydrogen number
q quarternary carbon
(br) broad signal

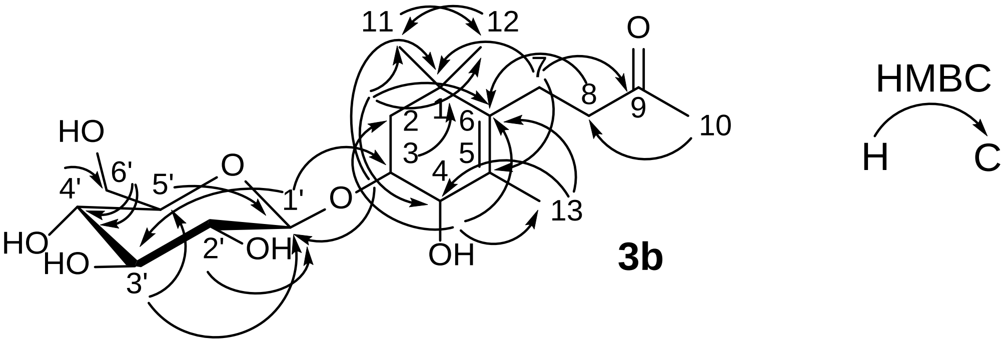

3b

Figure S4. Structure relevant long-range HC-correlation signals observed in the HMBC of 3b

Table S4. NMR data of 3,4-dihydroxy-7,8-dihydro-β-ionone 3-*O*-rutinoside (3c).[a]

Position[b]	δ_C ppm (CH_n)	δ_H ppm; mult., J Hz[c]	HMBC[d]
1	39.0 (C_q)		
2	40.4 (CH_2)	1.53-1.57 (a); m<br>1.87 (b); t, *J*=12.9 Hz	(a) 1,3,4,6<br>(b) 1,3,4,6,11,12
3	76.5 (CH)	3.83-3.89; m	2,1′
4	70.7 (CH)	3.95-3.99; m	2,3,5,6,13
5	128.2 (C_q)		
6	143.3 (C_q)		
7	23.1 (CH_2)	2.18-2.33; m	1,5,6,8,9
8	44.3 (CH_2)	2.58; t, *J*=8.3 Hz	6,7,9
9	211.4 (C_q)		
10	29.7 (CH_3)	2.14; s	8
11	27.7 (CH_3)	1.07; s	1,2,6,12
12	29.6 (CH_3)	1.05; s	1,2,6,11
13	18.3 (CH_3)	1.75; s	4,5,6
1′	103.2 (CH)	4.44; d, *J*=7.9 Hz	3′
2′	75.3 (CH)	3.18-3.23; m	3′,4′
3′	77.9 (CH)	3.34-3.38; m	1′,2′,4′
4′	71.6 (CH)	3.27-3.33; m	3′,5′,6′
5′	76.8 (CH)	3.39-3.43; m	3′,4′,6′
6′	68.0 (CH_2)	3.56-3.60 (a); m<br>3.95-3.99 (b); m	(a) 5′,1′′<br>(b) 4′,5′,1′′
1′′	102.1 (CH)	4.71; d, *J*=1.7 Hz	6′,2′′,3′′
2′′	72.4 (CH)	3.62-3.66; m	3′′
3′′	72.2 (CH)	3.81; dd, *J*=1.74/3.47 Hz	1′′,4′′
4′′	74.1 (CH)	3.34-3.38; m	2′′,5′′,6′′
5′′	69.8 (CH)	3.62-3.66; m	3′′
6′′	18.2 (CH_3)	1.25; d, *J*=6.1 Hz	4′′,5′′

[a] Solvent: CD_3OD; TMS (δ = 0.00 ppm) for ^1H, CD_3OD (δ = 49.00 ppm) for ^{13}C; ^1H observation frequency 600.1 MHz.
[b] For numbering of the carbon atoms, see the formula, assignment of C-H via HSQC data.
[c] For CH_2 groups with diastereotopic protons a and b indicate the deshielded and shielded nucleus, respectively.
[d] Entries in the column HMBC indicate ^1H nuclei showing long-range correlations ($^{2,3}J$) with the ^{13}C chemical shift in the second column.
n hydrogen number
q quarternary carbon

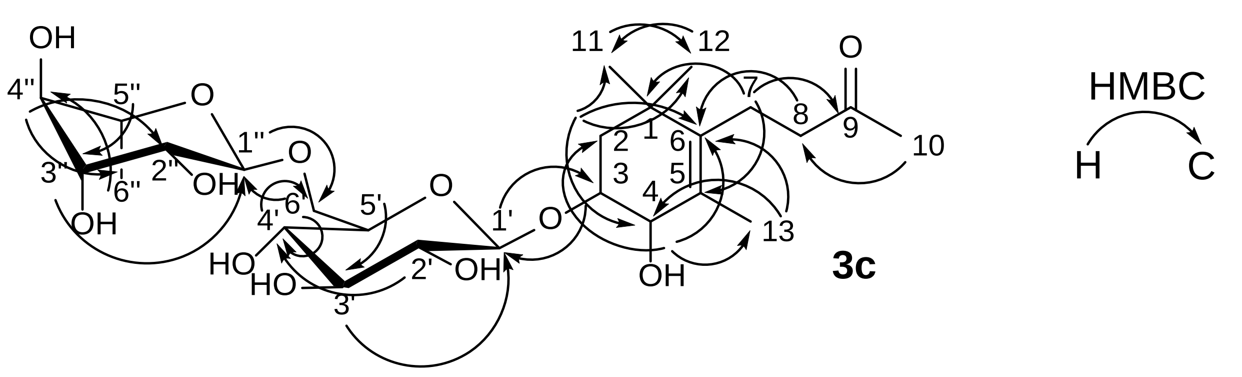

3c

Figure S5. Structure relevant long-range HC-correlation signals observed in the HMBC of 3c

References

Berthod, A.; Friesen, J.B.; Inui, T.; Pauli, G.F. Elution-extrusion countercurrent chromatography: theory and concepts in metabolic analysis, *Anal. Chem.* **2007**, 79, 3371-3382.

Ito, Y. Golden rules and pitfalls in selecting optimum conditions for high-speed countercurrent chromatography. *J. Chromatogr. A* **2005**, 1065, 145-168.

Stahl, E.; Kaltenbach, U. Dünnschichtchromatographie: VI. Mitteilung. Spurenanalyse von Zuckergemischen auf Kieselgur G-Schichten. *J. Chromatogr. A* **1961**, 5, 351-355.
